# Cottage Hospital Administration

**Published:** 1897-09-04

**Authors:** 


### COTTAGE HOSPITAL ADMINISTRATION.

### THE COMMITTEE AND MEDICAL STAFF.

The *Aberdeen Free Press* reports the proceedings at the meeting of the managers and the governors of the Huntly Cottage Hospital which reveal a state of friction between two members of the medical staff and the committee which is very regrettable. That friction has arisen because the Huntly Cottage Hospital was started without any rules for the admission of patients. This omission practically left the admission of patients in the hands of the medical staff. One of them took upon himself, without consulting the committee, to admit four cases of typhoid fever from an outlying district, the inhabitants of which did not contribute to its funds. Those patients ought to have been provided for by the local authority in a properly-organised infectious hospital, and we should like to know why Dr. Garson sent the patients to the Cottage Hospital, and failed to communicate with the local authorities, whose duty it is to provide suitable accommodation for this class of cases.

To prevent the occurrence of similar irregularities the committee drew up a number of rules, the chief of which enacted that no one not resident in the parishes contributing to the hospital should be considered eligible for admission unless in exceptional cases. Two of the medical staff, Dr. Garson and Dr. McRae, the chairman of the committee stated, were opposed to the rules, and refused to act under them. They did not, however, resign, as in the natural course they should have done, but took up a position hostile to the committee, which rendered the successful working of the Huntly Cottage Hospital difficult, if not impossible. The committee appear to have tried to induce the two medical officers to co-operate with them in enforcing necessary rules, and finding that they refused to alter their attitude, and that they did not resign, the committee summoned a meeting of the governors and placed their resignation in the hands of the meeting. In the result the meeting passed a vote of confidence in the Managing Committee and re-elected the whole of its members. Three of them, including the chairman, however, declined to serve again. The vacancies thus caused having been filled up, the proceedings terminated.

We think it is due to the subscribers to the Huntly Cottage Hospital, to the medical profession, and to their own reputation that Dr. Garson and Dr. McRae should give a full explanation of their conduct, or that they should resign their connection with the Huntly Cottage Hospital. Failing this the governors would be well advised to hold a special meeting and call on these gentlemen to resign. It is quite foreign to the spirit of cottage hospital management and to the interests of the patients and the public that a difficulty of the kind we have described should be allowed to continue in an acute form; and the sooner it is settled on a business basis the better it will be for the Huntly Cottage Hospital and for all who take an interest in its welfare and management.

